# DNA metabarcoding unveils multiscale trophic variation in a widespread coastal opportunist

**DOI:** 10.1111/mec.14886

**Published:** 2018-10-24

**Authors:** Andjin Siegenthaler, Owen S. Wangensteen, Chiara Benvenuto, Joana Campos, Stefano Mariani

**Affiliations:** ^1^ Ecosystems and Environment Research Centre School of Environment and Life Sciences University of Salford Salford UK; ^2^ CIIMAR Interdisciplinary Centre of Marine and Environmental Research University of Porto Matosinhos Portugal; ^3^ Present address: Department of Integrative Biology University of Guelph Guelph Canada; ^4^ Present address: Norwegian College of Fishery Science UiT the Arctic University of Norway Tromsø Norway

**Keywords:** community ecology, crustaceans, diet analysis, environmental DNA, food webs, metabarcoding

## Abstract

A thorough understanding of ecological networks relies on comprehensive information on trophic relationships among species. Since unpicking the diet of many organisms is unattainable using traditional morphology‐based approaches, the application of high‐throughput sequencing methods represents a rapid and powerful way forward. Here, we assessed the application of DNA metabarcoding with nearly universal primers for the mitochondrial marker cytochrome *c* oxidase I in defining the trophic ecology of adult brown shrimp, *Crangon crangon*, in six European estuaries. The exact trophic role of this abundant and widespread coastal benthic species is somewhat controversial, while information on geographical variation remains scant. Results revealed a highly opportunistic behaviour. Shrimp stomach contents contained hundreds of taxa (>1,000 molecular operational taxonomic units), of which 291 were identified as distinct species, belonging to 35 phyla. Only twenty ascertained species had a mean relative abundance of more than 0.5%. Predominant species included other abundant coastal and estuarine taxa, including the shore crab *Carcinus maenas* and the amphipod *Corophium volutator*. Jacobs’ selectivity index estimates based on DNA extracted from both shrimp stomachs and sediment samples were used to assess the shrimp's trophic niche indicating a generalist diet, dominated by crustaceans, polychaetes and fish. Spatial variation in diet composition, at regional and local scales, confirmed the highly flexible nature of this trophic opportunist. Furthermore, the detection of a prevalent, possibly endoparasitic fungus (*Purpureocillium lilacinum*) in the shrimp's stomach demonstrates the wide range of questions that can be addressed using metabarcoding, towards a more robust reconstruction of ecological networks.

## 1 INTRODUCTION

Trophic interactions provide important insights on a wide range of ecological dynamics, ranging from individual to ecosystem levels, which include animal behaviour, predator–prey interactions, food web structure and community ecology (e.g., Leray, Meyer, & Mills, 2015; Pinol, San Andres, Clare, Mir, & Symondson, 2014). The feeding strategy of key consumers can have pronounced influences on ecosystem dynamics (Hanski, Hansson, & Henttonen, 1991; Holling, 1965) and their stomach contents can reveal essential information on food item distribution and prey assemblage structure (Lasley‐Rasher, Brady, Smith, & Jumars, 2015). Crustaceans are a key component in marine/estuarine soft‐bottom habitats (Evans, 1983, 1984), and evaluating their diet is very challenging due to the complexity of direct observations on predation rates and the limitations associated with the identification of partially digested food items (Asahida, Yamashita, & Kobayashi, 1997; Feller, 2006; Symondson, 2002).

The recent application of high‐throughput sequencing (HTS) tools, such as metabarcoding, promises to revolutionize the way prey diversity and composition are estimated from gut contents or faeces of consumers (Kartzinel & Pringle, 2015; Leray et al., 2015). Metabarcoding refers to the identification of multiple taxa based on the screening of bulk DNA extracted from community or environmental samples (i.e., water, soil, faeces; Barnes & Turner, 2016), by means of massive parallel sequencing of PCR amplicons (Barnes & Turner, 2016; Taberlet, Coissac, Pompanon, Brochmann, & Willerslev, 2012a). Metabarcoding has proven to be highly effective for the identification of prey remains with improved taxonomic resolution, accuracy and speed of analysis, compared to traditional morphological methods (Berry et al., 2015; Casper, Jarman, Deagle, Gales, & Hindell, 2007; Symondson, 2002). Yet, some challenges remain, such as fragmentation of partially digested DNA, variability in taxon‐specific digestion rates, secondary predation and, typically, the presence of high proportion of DNA from the study organisms itself, which may reduce sequencing depth and render cannibalism undetectable (Barnes & Turner, 2016; Berry et al., 2015; Pinol et al., 2014). Furthermore, due to the sensitivity of these methods, in some cases, it might be difficult to discriminate between contaminant DNA and target DNA.

The brown shrimp, *Crangon crangon* (L.), is a key crustacean species in European coastal waters. Its wide distribution (i.e., from the White Sea to Morocco), year‐round occurrence and high abundance (>100 ind./m^2^; van der Veer, Feller, Weber, & Witte, 1998) make it an essential part of the coastal benthic food web (Ansell, Comely, & Robb, 1999; Campos & van der Veer, 2008; Evans, 1984), a major prey item for birds and fish (Evans, 1984; Walter & Becker, 1997), and an important target for fisheries, with recorded catches in 2011 up to 35,000 tons and more than 500 fishing vessels employed in the North Sea (Aviat, Diamantis, Neudecker, Berkenhagen, & Müller, 2011; Campos & van der Veer, 2008). The trophic position of *C. crangon* is still being discussed, being described as trophic generalist (Evans, 1983), carnivorous opportunist (Pihl & Rosenberg, 1984) omnivorous (Ansell et al., 1999; Raffaelli, Conacher, McLachlan, & Emes, 1989; Tiews, 1970) and probable scavenger (Ansell et al., 1999). As a juvenile, it relies mostly on the consumption of meiofaunal prey items while it switches to larger demersal organisms as an adult, including conspecifics and juvenile stages of several commercially important teleosts and bivalves (Evans, 1984; Oh, Richard, & Richard, 2001; Pihl & Rosenberg, 1984; van der Veer & Bergman, 1987; van der Veer et al., 1998). Previous studies showed considerable variation in prey item consumption, partly due to the brown shrimp's inherent trophic flexibility and niche breadth, but also because studies have relied on microscopic identification of prey remains (e.g., Boddeke, Driessen, Doesburg, & Ramaekers, 1986; Oh et al., 2001; but see also Nordström, Aarnio, & Bonsdorff, 2009); yet, prey items are usually macerated to a fine degree by *C. crangon*, and a high proportion of its stomach content is, consequentially, impossible to identify through morphological examination (Asahida et al., 1997; Wilcox & Jeffries, 1974). Furthermore, most studies on *C. crangon*'s diet to date have focused on a limited number of locations and relatively small spatial scales (e.g., Evans, 1984; Oh et al., 2001; Pihl & Rosenberg, 1984) while large‐scale studies are required to assess geographical variation in the shrimp's diet and to understand the relative importance of different prey items.

The degree to which food items are actively selected or passively ingested by consumers is an essential consideration in assessing the relative importance of different prey categories and understanding the trophic niche of consumers. Traditionally, indices are used to infer the predator's preference for prey based on the relative abundance of prey in the predator's diet and the prey's relative abundance in the environment (e.g., Peterson & Ausubel, 1984). Examples of commonly used indices are the Ivlev's electivity index (Ivlev, 1961) and the Jacob's index of selectivity (Jacobs, 1974), which also corrects for item depletion (Jacobs, 1974). Although some attempts have been made to link diet metabarcoding data with food availability in managed forests (Kowalczyk et al., 2011) and artificial mesocosms (Ray et al., 2016), no examples exist for wild marine animal trophic studies contrasted with whole‐community environmental DNA (eDNA) data.

Here, we report on a large‐scale analysis of the trophic ecology of *C. crangon*, which reveals its ecological role in estuarine systems and provides a key for the reconstruction of ecological networks of European coastal marine communities. By using nearly universal primers for mitochondrial cytochrome *c* oxidase I, we used metabarcoding to describe the diet of the shrimp, alongside the soft‐bottom communities on which they feed, over a European scale. We were expecting a wide variety of prey items, reflecting variation in environmental conditions and prey availability across European coasts. More specifically, we tested whether metabarcoding can (a) provide a detailed overview of *C. crangon*'s diet, including prey selectivity, using DNA extracted from stomach and environmental samples; (b) identify geographical patterns in its trophic ecology, at both local and regional scales; and (c) assess consistent and general trophic patterns in order to better define the ecological role of this widespread species.

## 2 METHODS

### 2.1 Sample collection and processing

**Figure 1:**
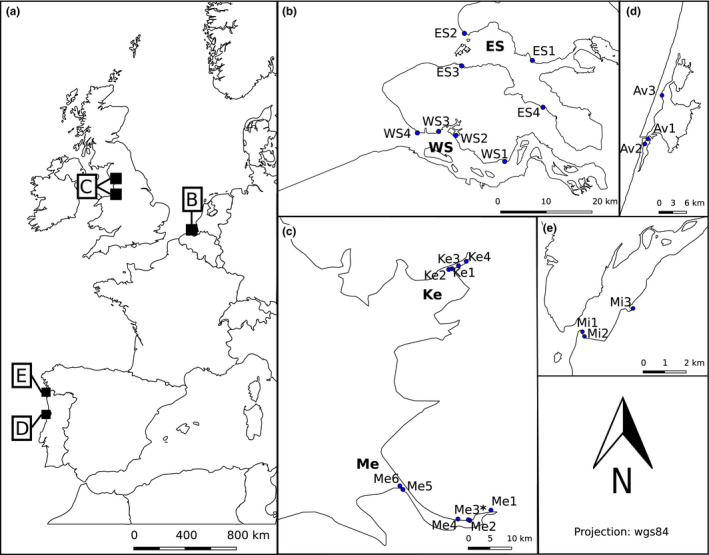
Overview of sample locations, illustrating (a) the overall western European scale; (b) the Dutch estuaries, Western Scheldt (WS) and Eastern Scheldt (ES); (c) the British estuaries, Mersey (Me) and Kent (Ke); (d) the Aveiro Ria (Av) and (e) the Minho estuary (Mi) in Portugal. Small dots within estuaries represent individual collection points for shrimp and sediment samples. *Site removed prior to molecular analysis. Source map: OpenStreetMap [Colour figure can be viewed at wileyonlinelibrary.com]

Brown shrimp and sediment samples were collected from 24 sites distributed over six estuaries in the Netherlands, Portugal and the United Kingdom (Figure 1). Adult shrimp (>20 mm total length, TL; tip of the rostrum to tip of the telson) were captured in the intertidal zone (0–1 m depth) by push‐net at low tide (±3 hr). Shrimp (30–50 per site) were placed on ice and transported to the laboratory to be stored at −20°C. Sediment was collected for the extraction of eDNA to characterize the biological community present at each site. Sediment was sampled from the upper 2‐cm surface layer, which represents the most recent DNA deposits and the habitat where the shrimp live and feed (Pinn & Ansell, 1993; Turner, Uy, & Everhart, 2015), with a PVC corer (3.2 mm Ø). Per site, three sediment subsamples were collected at several metres distance from each other and combined to reduce the influence of local heterogeneity (Taberlet et al., 2012b). The sediment was stored in 96% ethanol, transported on ice and kept at −20°C. At each site, temperature, salinity (Fisher Scientific Traceable Salinity Meter), pH (Hanna HI 98129), dissolved oxygen (OxyGuard Handy Mk I) and turbidity (Eutech TN‐100) were measured in triplicates. Extra sediment was collected, in triplicates, from each site for granulometric analyses (Horiba LA‐950 Particle size analyser) and total organic matter (TOM) determination by means of ashing (550°C, 6 hr). One site (Mersey 3) was not included for analysis because HTS of its stomach samples did not result in sufficient read depth (<1,000 reads; see Section 2.1).

### 2.2 DNA extraction

Overall, 1,025 shrimp (20–50 mm TL) were caught and 494 full stomachs (visual determination) were dissected using flame‐sterilized tools to avoid cross‐contamination. Stomachs were pooled in batches of eight (from shrimp collected at the same site) prior to DNA extraction (Ray et al., 2016). Though the pooling of samples increases the number of stomachs analysed per sequencing run, pooling might reduce the detected molecular taxonomic unit (MOTU) richness since large, recently ingested diet items in single stomachs may obscure the stomach contents of other individuals. Nevertheless, this potentially negative effect of pooling was deemed to be negligible, due to the pool replication conducted within location, and the population emphasis of the study. Three replicate pools were extracted per site. However, due to a high percentage of empty stomachs in natural populations (20%–60%; Feller, 2006; Oh et al., 2001; Pihl & Rosenberg, 1984), some sites contained only two replicates and some replicates contained less than eight full stomachs (see Supporting Information Table S1): the latter were still included in the analyses as variation in number of stomachs pooled did not affect the patterns observed (see Section 2.1). In total, 66 pooled samples were extracted, divided over 24 sites. In addition to the full stomach samples, three pooled samples of eight visually empty stomachs were included for comparative purposes.

DNA was extracted from 0.25 g of homogenized pooled stomach contents (*N* = 66) using the PowerSoil^®^ DNA Isolation Kit (Mo‐Bio laboratories), whereas DNA from sediment (10 g; *N* = 24) was extracted using the PowerMax^®^ DNA Soil Kit (Mo‐Bio laboratories). A Qubit fluorometer (Thermo Fisher Scientific) was used to assess DNA concentrations of purified extracts. DNA extraction and pre‐PCR preparations were performed in separate laboratories from post‐PCR procedures to reduce contamination.

### 2.3 DNA amplification and high‐throughput sequencing

Amplification of DNA, for both stomach and sediment samples, was achieved using a single set of versatile, highly degenerated PCR primers targeting the 313‐bp Leray fragment (Leray et al., 2013) of the mitochondrial cytochrome *c*. oxidase subunit I (COI) region. The mICOIintF‐XT primer (5′‐GGWACWRGWTGRACWITITAYCCYCC‐3′) was used as forward primer. This modified version (Wangensteen, Palacín, Guardiola, & Turon, 2018) of the mlCOIintF primer (Leray et al., 2013) included two extra degenerate bases (equimolar mixtures of two different bases at a given position) and two inosine nucleotides (that can match any nucleotide) to enhance its eukaryotic universality. The reverse primer was jgHCO2198 (5′‐TAIACYTCIGGRTGICCRAARAAYCA‐3′; Geller, Meyer, Parker, & Hawk, 2013). The Leray fragment has already been successfully applied for both the characterization of marine communities and marine fish gut contents (Leray & Knowlton, 2015; Leray et al., 2013, 2015). Eight‐base oligo‐tags (Coissac, Riaz, & Puillandre, 2012) attached to the metabarcoding primers were added to the amplicons during a single PCR step, in order to label different samples in a multiplexed library. Also, a variable number (2, 3 or 4) of fully degenerate positions (Ns) was added at the beginning of each primer, in order to increase variability of the amplicon sequences (Guardiola et al., 2015). The PCR mix recipe included 10 μl AmpliTaq gold 360 Master mix (Applied Biosystems), 3.2 μg bovine serum albumin (Thermo Scientific), 1 μl of each of the 5 μM forward and reverse tagged primers, 5.84 μl H_2_O and 2 μl extracted DNA template (~5 ng/μl). The PCR profile included an initial denaturing step of 95°C for 10 min, 35 cycles of 94°C for 1 min, 45°C for 1 min and 72°C for 1 min and a final extension step of 72°C for 5 min. After quality check of all amplicons by electrophoresis, the tagged PCR products (including two PCR‐negative controls) were pooled at equimolar concentration into two multiplexed sample pools (sediment and stomach) and purified using MinElute columns (Qiagen). Two Illumina libraries were subsequently built from these pools, using the NextFlex PCR‐free library preparation kit (BIOO Scientific). Libraries were quantified using the NEBNext qPCR quantification kit (New England Biolabs) and pooled in a 1:4 sediment:stomach molar concentration ratio (similar to the sediment:stomach sample ratio) along with 0.7% PhiX (v3, Illumina) serving as a positive sequencing quality control. The libraries with a final molarity of 8 pM were sequenced on an Illumina MiSeq platform using v2 chemistry (2 × 250 bp paired‐ends).

Preliminary analyses of the sequencing data revealed a substantial number of reads belonging to one MOTU in the fungal order Hypocreales (Ascomycota). For further identification, the internal transcribed spacer fragment was amplified from five samples with a high number (>90% read abundance) of reads of this MOTU, with the primer combination ITS1f (5′‐CTTGGTCATTTAGAGGAAGTAA‐3′; Gardes & Bruns, 1993) and ITS4ASCO (5′‐CGTTACTRRGGCAATCCCTGTTG‐3′; Nikolcheva & Bärlocher, 2004), specific for Ascomycota. The PCR mix recipe was the same as the one used for the Leray fragment described above and the PCR profile included an initial denaturing step of 95°C for 5 min, 32 cycles of 95°C for 30 s, 55°C for 30 s and 72°C for 1 min and a final extension step of 72°C for 10 min (Manter & Vivanco, 2007). After electrophoresis check, the amplicons of these five samples were cleaned and Sanger sequenced by Source Bioscience Sequencing UK.

### 2.4 Bioinformatic and data analyses

Bioinformatic analyses were performed using the obitools metabarcoding software suite (Boyer et al., 2016). Read quality assessment was performed with FastQC and paired‐end read alignment using illuminapairedend, retaining reads with an alignment quality score >40. Demultiplexing and primer removal were achieved using ngsfilter with the default options. Obigrep was applied to select all aligned reads with a length between 303 and 323 bp and free of ambiguous bases. Obiuniq was used to dereplicate the reads, and the uchime‐denovo algorithm (Edgar, Haas, Clemente, Quince, & Knight, 2011) (implemented in VSEARCH; Rognes, Flouri, Nichols, Quince, & Mahe, 2016) was used to remove chimeras. Amplicon clustering was performed using the SWARM algorithm (Mahé, Rognes, Quince, de Vargas, & Dunthorn, 2014, 2015) with a d value of 13, which offers a conservative solution to the high variability of the COI gene (Wangensteen & Turon, 2017). After removal of singletons, taxonomic assignment of the representative sequences for each MOTU was performed using the ecotag algorithm (Boyer et al., 2016), using a local reference database (Wangensteen et al., 2018) containing filtered COI sequences retrieved from the bold database (Ratnasingham & Hebert, 2007) and the EMBL repository (Kulikova et al., 2004). This algorithm uses a phylogenetic approach to assign sequences to the most reliable monophyletic unit, based on the density of the reference database. The data were refined by clustering MOTUs assigned to the same species, abundance renormalization (to remove false positives due to tag‐switching; Wangensteen & Turon, 2017) and by removing bacterial reads and contaminations of human or terrestrial origin. MOTUs with a maximum of four or less reads per sample were removed on a sample by sample basis to avoid false positives and low‐frequency noise (De Barba et al., 2014; Wangensteen et al., 2018). All MOTUs for which the abundance in the PCR‐negative controls was higher than 10% of the total reads of that MOTU were removed (Wangensteen & Turon, 2017). Samples with a low read depth (<1,000) following removal of predator, parasite and contaminant reads were removed prior to analysis. All statistical analyses were performed in *R* version 3.1.3 (https://www.R-project.org/) with the vegan (version 2.3‐5) and biodiversityr (version 2.5‐3) packages (Kindt & Coe, 2005; Oksanen et al., 2016). Only MOTUs showing mean relative abundance ≥0.5% in the full stomach samples were considered (Albaina, Aguirre, Abad, Santos, & Estonba, 2016) for nonmetric multidimensional scaling (nMDS), canonical correspondence analysis (CCA) and PERMANOVAs. Correlation between sediment and stomach community composition was tested with a Mantel test (Bray–Curtis dissimilarities; Pearson's product–moment correlation; 999 permutations). The influence of environmental variables (mean temperature, salinity, pH, oxygen saturation, turbidity, median sediment grain size and TOM) on the full stomach contents were tested by means of CCA and PERMANOVA. PERMANOVAs were calculated using the function Adonis (vegan) with Bray–Curtis dissimilarities and 1,000 permutations. Prior to CCA and PERMANOVA, model selection was performed using the function ordistep (vegan). Prey MOTU richness for each estuary was represented as MOTU accumulation curves after rarefaction for the number of reads (1,000 reads, 500 permutations) and the number of samples (9–15 samples, 1,000 permutations). The Jacobs’ selectivity index was calculated based on the relative read abundances of the MOTUs extracted from sediment and stomach samples in accordance with Jacobs (1974). Trophic significance of individual MOTU was determined based on the relative read abundance, fraction of samples with MOTU presence and Jacobs’ selectivity index as follows: Trophic significance = (relative abundance) * (fraction of samples) * (Jacobs’ selectivity index + 1). Trophic significance was represented in categorical terms based on the relative trophic significance of each MOTU (high: >10%, medium 1%–9%, low <1%) instead of exact values since the relative abundances of individual taxa should be considered with caution (Deagle et al., 2005).

## 3 RESULTS

### 3.1 Collection statistics

A total of 1,025 *Crangon crangon* were caught with a 1:8 male:female sex ratio (based on 767 shrimp which could be sexed morphologically). About 7.5% of the females were ovigerous. Mean (±*SD*) wet weight was 0.40 ± 0.26 g; mean (±*SD*) TL was 35.1 ± 7.6 mm; and mean (±*SD*) carapace length (CL) was 7.4 ± 1.6 mm (CL = 0.214*TL; *r* ^2^ = 0.81, *N* = 1,025). TL varied significantly between sites (Supporting Information Table S2; one‐way ANOVA: *df* = 23, *F* = 47.95, *p* < 0.001). Overall, the proportion of *C. crangon* with a full stomach was 57.9%. Mean proportion of full stomachs per site (58.9 ± 19.3%) was not correlated with the time of sampling (Pearson's correlation: *R* ^2^ = 0.07, *p* = 0.754, *N* = 24).

### 3.2 High‐throughput DNA sequencing

**Figure 2:**
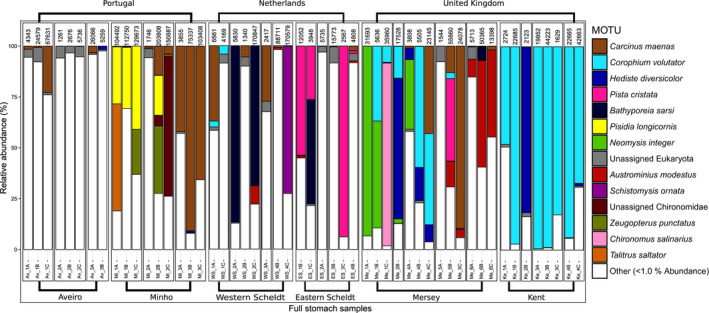
Relative abundances of MOTUs detected in *Crangon crangon* stomach samples by COI metabarcoding, after removal of *C. crangon* and *Purpureocillium lilacinum* reads. Each bar represents one sample. Countries are shown on top of the graph, estuaries below and boxes contain the individual sites. The number on top of each sample represents the number of diet‐related COI reads. The category "other" is comprised of MOTUs with <1.0% COI reads

**Figure 3:**
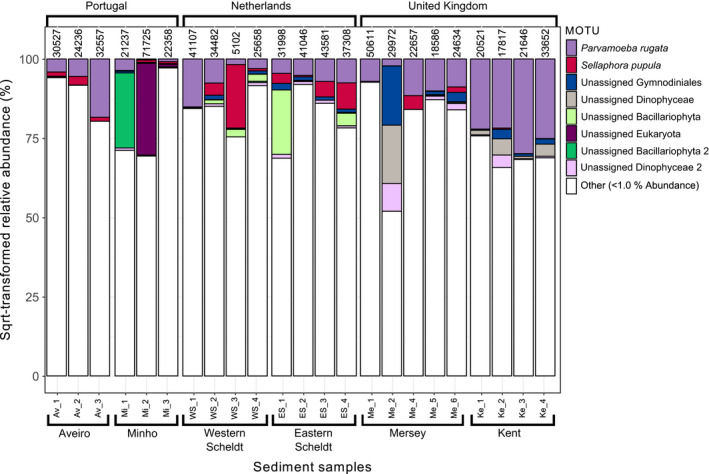
Relative abundances of MOTUs detected in sediment samples by COI metabarcoding. Each bar represents one sample. Countries are shown on top of the graph, estuaries below. The number on top of each sample represents the number of COI reads. The category “other” is comprised of MOTUs with <1.0% COI reads

**Figure 4:**
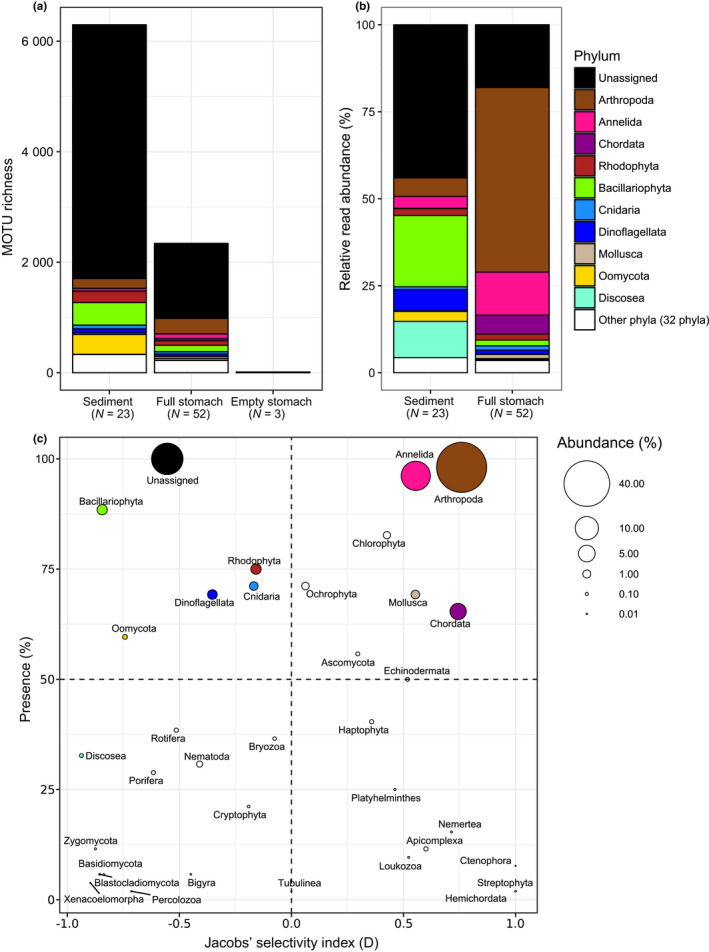
Phyla detected in sediment and *Crangon crangon* stomach samples by COI metabarcoding. (a) Total number of MOTU detected per phylum in sediment, full stomachs and visually empty stomachs. (b) Mean relative read abundance of phyla detected in sediment and *C. crangon* full stomach samples, after removal of *C. crangon* and *Purpureocillium lilacinum* reads. (c) Phylum trophic significance based on presence (%), mean relative abundance (%) in full stomach samples and Jacobs’ selectivity index. Stomach samples consisted of a pool of up to eight stomachs. The category “other phyla” (represented in white) contains phyla with <1% COI reads in both the sediment and full stomach samples

A total number of 8,895,448 reads were obtained from an Illumina MiSeq run of pooled amplicon libraries built from 24 sediment samples, 69 pooled *C. crangon* full stomach samples (from now on referred to as stomach samples), three pooled *C. crangon* empty stomach samples (comprising of stomach tissue and clear liquid) and two PCR‐negative controls. Variation in the number of pooled stomachs did not affect the patterns of diet composition (PERMANOVA: pseudo‐*F*_1,50_ = 1.0, *p* = 0.453) or MOTU richness (rarefied to 1,000 reads) per sample (generalized linear model with quasipoisson distribution: *t* = 1.08, *p* = 0.650). In total, 5,704,471 reads remained after sample demultiplexing, quality and sequence‐length filtering, and removal of bacterial reads, contaminations and false positives due to tag‐switching (sediment samples: 742,286; stomach samples: 4,828,136; empty stomach samples: 134,049). After taxonomic assignment, a total of 39 MOTUs (16 Metazoa, seven Rhodophyta, five Stramenopiles, 11 unassigned) were removed because their abundance in the PCR‐negative control was >10% of the total reads of those MOTUs. Mean (±*SD*) proportion of *C. crangon* reads was 28 ± 29% (range: 0.2%–97.6%) in the stomach samples and 47 ± 46% (range: 10.6%–99.1%) in the empty stomach samples. Mean proportion of *C*. *crangon* reads was 1 ± 4% in the sediment samples (range: 0.0%–21.0%). Remaining number of reads per sample ranged 179–203,808 in full stomach, 7–332 in empty stomach and 5,114–71,770 in sediment samples. A high number of reads (4,828,136 reads) belonging to a fungus of the species *Purpureocillium lilacinum* (Ascomycota: Hypocreales) were detected in almost all (95%) stomach samples and identified using both COI (100% identity) and ITS markers (100% identity; Supporting Information Table S3). Mean (±*SD*) proportion of *P. lilacinum* reads was 36 ± 37% (range: 0.0%–97.4%) in full stomach, 53 ± 47% (range: 0.1%–89.4%) in empty stomach and 0.1 ± 0.2% (range: 0.0%–0.8%) in sediment samples. No *P. lilacinum* were detected in the PCR‐negative controls. All *C. crangon* and *P. lilacinum* reads were removed from the database prior to further analyses on diet, resulting in a total of 2,687,877 reads divided over 66 pooled stomach samples (Figure 2) and 24 sediment samples (Figure 3). A total of 14 pooled stomach samples were removed, prior to further analyses, because they contained less than 1,000 diet‐related reads (Supporting Information Table S4). One sediment sample (Mersey 3) was, consequently, also removed since no stomach samples were included for that site. The final data set consisted of a total of 8,321 MOTUs, of which 6,299 MOTUs belonging to 40 phyla were detected in the sediment samples, 2,342 (35 phyla) in the stomach samples, and 14 (seven phyla) in the empty stomach samples. A total of 502 MOTUs were detected both in the sediment and in stomach samples and only two (an unassigned Rhodophyta and an unassigned Eukaryota) were detected exclusively in the empty stomach samples. Of the total number of MOTUs detected, 370 could be assigned to the species level of which 291 were detected in the stomach samples. Twenty taxa showed a relative abundance greater than 5% in any given sample while 85 taxa showed an abundance greater than 1%. The final number of diet‐related reads per stomach sample varied randomly, without systematic trends across estuaries (one‐way ANOVA: Estuary: *F*_5,29_ = 2.017, *p* = 0.106; Sites nested in estuary: *F*_17,29_ = 0.811, *p* = 0.669). Rarefaction curves (Supporting Information Figure S1) showed that a plateau in the number of MOTUs was achieved in almost all cases, indicating an overall sequencing depth adequate to capture the number of MOTUs present. Empty stomach samples contained a very low number of MOTUs and reads and were, therefore, not taken into account for any further analyses (Figure 4a).

### 3.3 Description of *Crangon crangon* diet

Analysis of *C. crangon* stomach contents showed large variation in relative MOTU abundances between samples (Figure 2). Notable patterns are the lack of a dominant MOTU detected in stomachs from the Aveiro Ria; a relatively high (10%–25%) contribution of the decapod crabs *Carcinus maenas* and *Pisidia longicornis* in the Minho estuary; the detection of the introduced barnacle *Austrominius modestus* in the Scheldt and Mersey estuaries; high amounts (~25%) of the polychaete *Pista cristata* in the Eastern Scheldt; the substantial proportion (~10%) of the mysid *Neomysis integer* reads in the Mersey estuary, and the dipteran *Chironomus salinarius* also, in the most inland sampling site of this estuary (>75%); the large contribution (~50%) of the amphipod *Corophium volutator* in the Kent estuary. In general, the shore crab *C. maenas* and the amphipod *C. volutator* were the trophically preponderant prey items for *C. crangon* (Table 1). Other important MOTUs included annelids (*Hediste diversicolor* and *P. cristata*), other amphipods (*Bathyporeia sarsi*), other decapods (*P. longicornis*), chironomids (unassigned), mysids (*N. integer*) and barnacles (*A. modestus*). Fish reads were detected in all estuaries with a total of 22 species present in 27 stomach samples. Five fish species were relatively abundant (≥5%; Table 1) but were generally only present in a small number of stomach samples. One other noteworthy observation is the presence of low abundances of known parasitic taxa in several stomach samples (*Hematodinium* sp., three stomach samples; Apicomplexa, six stomach samples).

**Table 1:** Trophic significance of *Crangon crangon* prey items

Phylum	Order	Family	Species	Best identity	Presence (%)	Mean (±*SE*) abundance (%)	Mean (±*SE*) selectivity (D)	Trophic significance	Literature	Source
Annelida	Phyllodocida	Nereididae	*Hediste diversicolor*	0.98	30.8	7.1 ± 4.5	0.9 ± 0.0	Medium	Sto+	Lloyd and Yonge (1947), Pihl and Rosenberg (1984)
	Spionida	Spionidae	*Scolelepis foliosa*	1.00	5.8	0.7 ± 0.6	1.0 ± 0.0	Low	Exp^a^ Sto−a	Van Tomme, Degraer and Vincx (2014), Ansell et al. (1999)
	Terebellida	Terebellidae	*Pista cristata*	0.99	21.2	4.5 ± 2.6	0.6 ± 0.2	Medium	Sto−a	Ansell et al. (1999)
Arthropoda	**Amphipoda**	**Corophiidae**	***Corophium volutator***	**0.99**	**28.8**	**13.8 ± 5.9**	**1.0 ± 0.0**	**High**	Sto+	Pihl and Rosenberg (1984), Evans (1984)
		Pontoporeiidae	*Bathyporeia sarsi*	1.00	7.7	3.5 ± 2.5	1.0 ± 0.0	Medium	Exp	Van Tomme et al. (2014)
		Talitridae	*Talitrus saltator*	0.99	1.9	0.8 ± 0.8	1.0 ± 0.0	Low		
	Calanoida	Centropagidae	*Centropages typicus*	1.00	5.8	0.7 ± 0.7	1.0 ± 0.0	Low		
	**Decapoda**	**Carcinidae**	***Carcinus maenas***	**1.00**	**55.8**	**8.0 ± 3.3**	**1.0 ± 0.0**	**High**	Exp^b^ Sto−	Moksnes et al. (1998), Raffaelli et al. (1989), Pihl and Rosenberg (1984)
		Porcellanidae	*Pisidia longicornis*	1.00	9.6	1.7 ± 1.5	1.0 ± 0.0	Medium		
	Diptera	Chironomidae	Unassigned	0.88	53.8	0.9 ± 0.4	0.8 ± 0.1	Medium	CA	Nordström et al. (2009)
		Chironomidae	*Chironomus salinarius*	1.00	1.9	1.3 ± 1.3	1.0 ± 0.0	Low		
	Mysida	Mysidae	*Mesopodopsis slabberi*	0.99	3.8	0.6 ± 0.6	1.0 ± 0.0	Low		
		Mysidae	*Neomysis integer*	0.98	13.5	2.7 ± 2.1	1.0 ± 0.0	Medium	Sto−	Raffaelli et al. (1989)
		Mysidae	*Schistomysis ornata*	0.98	1.9	1.6 ± 1.6	1.0 ± 0.0	Low	Sto+^a^	Oh et al. (2001)
	Sessilia	Austrobalanidae	*Austrominius modestus*	1.00	17.3	2.0 ± 1.5	1.0 ± 0.0	Medium		
Chordata	Atheriniformes	Atherinidae	*Atherina presbyter*	1.00	1.9	0.6 ± 0.6	1.0 ± 0.0	Low		
	Clupeiformes	Clupeidae	*Sardina pilchardus*	1.00	3.8	0.6 ± 0.6	1.0 ± 0.0	Low		
	Pleuronectiformes	Scophthalmidae	*Zeugopterus punctatus*	0.99	3.8	0.8 ± 0.6	1.0 ± 0.0	Low		
	Scombriformes	Scombridae	*Scomber scombrus*	1.00	1.9	1.0 ± 1.0	1.0 ± 0.0	Low		
	Spariformes	Sparidae	*Spondyliosoma cantharus*	1.00	7.7	0.6 ± 0.4	1.0 ± 0.0	Low		
Cnidaria	Actiniaria	Actiniidae	*Anthopleura elegantissima*	0.99	3.8	0.6 ± 0.6	1.0 ± 0.0	Low		

*Notes*

MOTUs shown (≥0.5% mean relative abundance in the stomach samples) are assigned to the family level or lower.

CA: contribution assumed by source; Exp: Experimental study; Sto+: major contributor based on stomach analysis; Sto−: minor contributor based on stomach analysis. In bold: High trophic significant taxa. Best identity: alignment score of the best match in the reference database.

^a^Related taxa (same family); ^b^Larvae.

### 3.4 Selectivity in *Crangon crangon* diet

**Figure 5:**
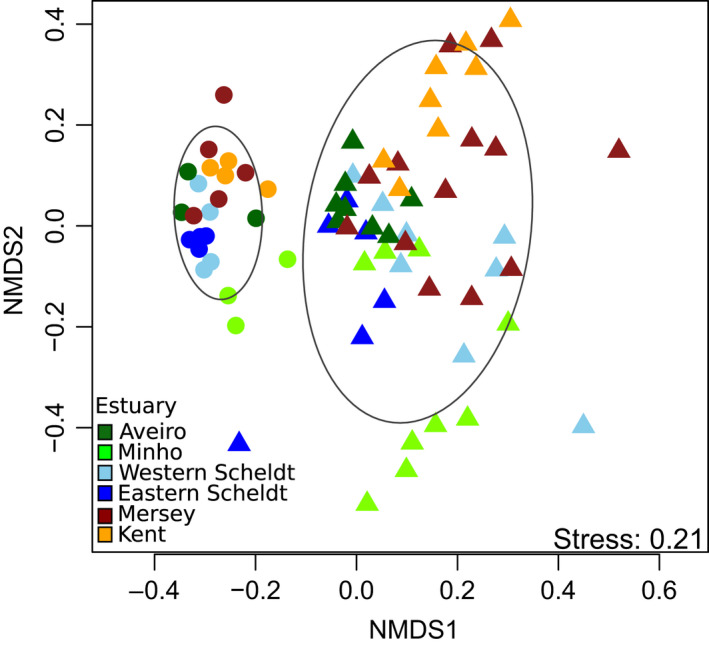
Multidimensional scaling analysis of MOTUs detected in sediment (dots) and *Crangon crangon* stomach samples (triangles), based on square‐root‐transformed Bray–Curtis dissimilarities. 75% confidence ellipses are shown per sample type

**Figure 6:**
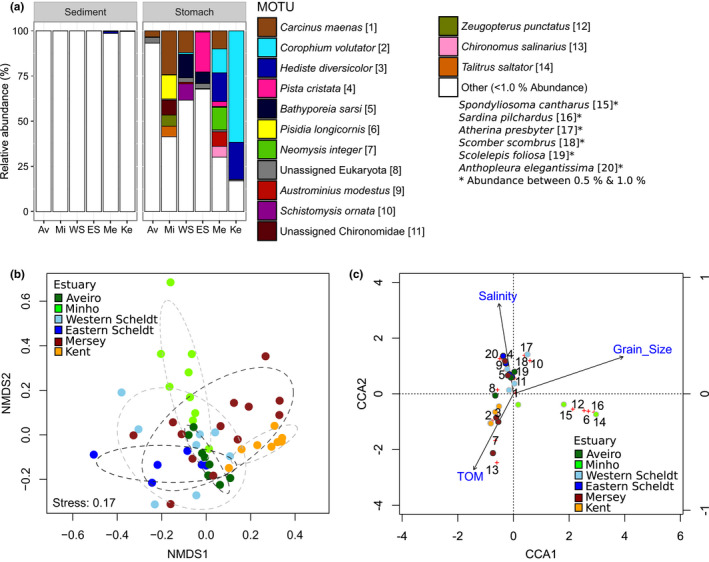
Multivariate analysis of *Crangon crangon* diet in six estuaries determined by COI metabarcoding based on MOTUs (*N* = 20) over all stomach samples (*N* = 53). (a) Mean relative read abundance of each MOTU per estuary based on DNA extracted from sediment and stomach samples (after removal of *C. crangon* and *Purpureocillium lilacinum* reads). MOTUs are identified for ≥1.0% average read abundance in the stomach samples, otherwise are referred as “Other”. (b) Nonmetric multidimensional scaling (nMDS) analysis based on Bray–Curtis dissimilarities of square‐root‐transformed relative abundances in *C. crangon* stomach samples. Each dot represents one pooled stomach sample, estuaries are identified by colours (see below), and ellipses show 75% confidence intervals. (c) Canonical correspondence analysis (CCA) of square‐root‐transformed relative read abundances in relation to salinity, total organic matter (TOM) and median grain size. Reads were averaged per site (displayed as dots) and estuaries are identified by colour (see below). Red crosses represent the MOTU scores and numbers refer to the MOTU names given in panel (a)

MOTU diversity within phyla was generally higher in the sediment than in the stomach samples, except for Arthropoda, Annelida, Mollusca and Chordata (Figure 4a). The proportion of MOTUs that could not be assigned at the phylum level was higher in the sediment (73%) than in the stomach samples (58%), and many abundant taxa in the sediment could not be identified at lower taxonomic ranks (Figure 3). Data combined per sample type (sediment/stomach) and MOTUs pooled at the phylum level showed that sediment samples contained high relative read abundances of Bacillariophyta (20 ± 3%), Discosea (10 ± 2%), Dinoflagellata (6 ± 2%) and Arthropoda (5 ± 1%) while *C. crangon* stomach samples contained a high mean (±*SE*) relative read abundance (%) for Arthropoda (53 ± 5%), Annelida (12 ± 3%) and Chordata (5 ± 2%; Figure 4b). Mantel test results showed a significant correlation between the community structure detected in the stomach and sediment samples (*r*: 0.43, *p* < 0.01), indicating an association between the shrimp's diet and its prey abundance in the environment. Analyses of DNA extracted from both sediment and *C. crangon* pooled stomach samples showed, based on all MOTUs detected, significant differences between sample types and estuaries (Figures 5 and 6a; PERMANOVA: sample type: pseudo‐*F*_1,68_ = 7.8, *p* < 0.001; estuary: pseudo‐*F*_5,68_ = 2.5, *p* < 0.001). Visual inspection of the relative abundances of the most important MOTUs also showed a high discrepancy between the abundances in the stomach (Figure 2) and sediment samples (Figure 3). MOTUs abundant in the stomach samples (≥1% abundance) showed, furthermore, a low read abundance in the sediment samples in all estuaries (Figure 6a). These differences in relative abundances resulted in many MOTUs having a maximum Jacobs’ selectivity index value of one which indicates that prey items were highly selected (Table 1). Phylum composition differed significantly between sediment and full stomach samples (PERMANOVA: pseudo‐*F*_1,44_ = 34.1, *p* < 0.001). Apart from Cnidaria and Rhodophyta, all phyla with ≥1% abundance in either sediment or stomach samples showed significant differences (based on paired Wilcoxon signed‐rank tests) in relative read abundances between the sediment and stomach samples (Supporting Information Table S5). Visualization of the importance of the phyla detected in the stomach samples based on the mean relative abundance (%), presence (%) and Jacobs’ selectivity index (D) is shown in Figure 4c.

### 3.5 Variation between estuaries

**Figure 7:**
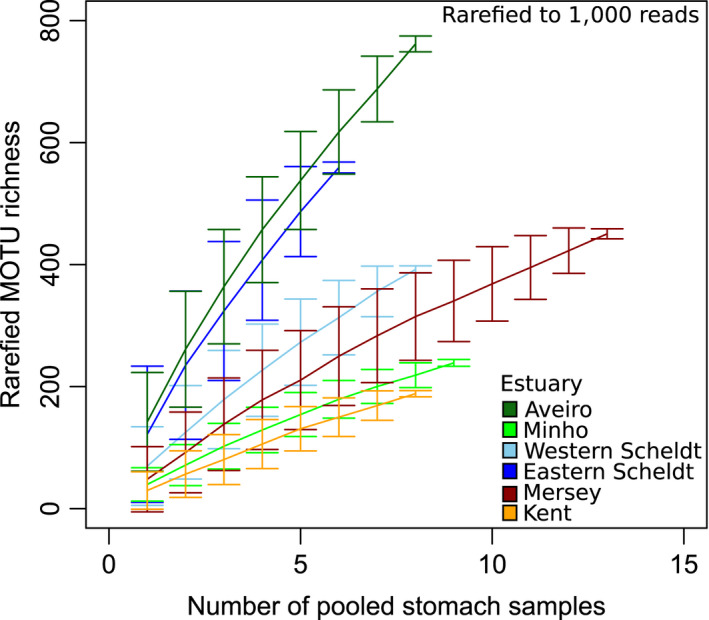
MOTU accumulation curves showing MOTU richness (based on all MOTUs detected) in *Crangon crangon* pooled stomach samples in several European estuaries. Each sample has been rarefied to 1,000 reads prior to the construction of the accumulation curves. Stomach samples consisted of a pool of up to eight stomachs

Multivariate analysis on the stomach contents (MOTUS ≥0.5% abundance) showed significant differences between estuaries (Figure 6b; PERMANOVA: pseudo‐*F*_5,29_ = 2.7, *p* < 0.001) and sites nested within estuaries (PERMANOVA: pseudo‐*F*_17,29_ = 0.6, *p* < 0.001). Bonferroni‐corrected pairwise comparisons showed similarity in consumed community structure among the Eastern Scheldt, Western Scheldt and Mersey estuaries. Minho differed significantly from the Mersey and Eastern Scheldt estuaries. Aveiro or Kent significantly differed from all other estuaries (See Supporting Information Table S6 for details). Step‐wise model selection (both forward and reverse) and CCA (Figure 6c) showed significant influences of salinity (*p* < 0.01), median grain size (*p* < 0.01) and TOM (*p* < 0.05; see Supporting Information Table S7 for means per estuary) on MOTU composition in *C. crangon* stomach samples (≥0.5% abundant MOTUs). The environmental variables (constrained CCA axes) explained 34% of the variance in the data set. Temperature, turbidity and oxygen saturation did not have a significant influence on the model, and pH was strongly correlated with salinity (*r* ^2^ = 0.75, *p* < 0.001, *N* = 23). These factors were, therefore, not included in the final model. MOTU richness (rarefied to 1,000 reads) in *C. crangon* stomach contents also showed differences between estuaries, with the Aveiro and Eastern Scheldt estuaries showing a higher number of MOTUs than the others (Figure 7). The slopes of the MOTU accumulation curves, however, did not approach an asymptote, offering a glimpse of the vast amount of marine biodiversity yet to be uncovered.

## 4 DISCUSSION

### 4.1 Evaluation of *Crangon crangon* diet

This study provides a detailed overview of the brown shrimp's trophic ecology, focusing on dietary variations at multiple geographical scales. Adult brown shrimp were caught in a variety of sandy estuarine intertidal habitats. Mainly females were captured, probably due to the spatial sex‐specific segregation of *C. crangon* during the summer–autumn period (Bamber & Henderson, 1994; Henderson & Holmes, 1987). The results confirm *C. crangon* as a generalist consumer feeding on a broad variety of food items but preferring larger mobile epifaunal prey items such as crustaceans, annelids and fish. The present investigation uncovered a great diet contribution of decapods and teleosts, while these were usually not considered to be important contributors to the shrimp diet in previous studies (e.g., Ansell et al., 1999; Oh et al., 2001; Plagmann, 1939; Raffaelli et al., 1989). Although comparisons of prey contribution should be made cautiously due to diversity of quantification methods used, the observed trend could be partly explained by scavenging behaviour on large organisms, previously not recorded in crangonid shrimps. Crangonid shrimps generally macerate and eat the soft body parts of larger preys (Asahida et al., 1997; Gibson, Yin, & Robb, 1995; Seikai, Kinoshita, & Tanaka, 1993; Wilcox & Jeffries, 1974). Smaller food items, on the other hand, are often ingested as a whole, including their hard body parts (Tiews, 1970), and are thus more easily identified by morphological methods. This discrepancy in detectability might possibly have played a role in studies that have detected low amount of fish and decapods but considerable amounts of unidentified soft tissue (e.g., Oh et al., 2001; Raffaelli et al., 1989). Metabarcoding methods can detect and taxonomically identify such soft tissues, thus highlighting the enhanced suitability of molecular approaches to present a more realistic picture of trophic ecology in marine invertebrates.

The diet of *C. crangon* showed a high MOTU richness, including previously described food items (Table 1). The number of COI MOTUs (2,342) detected in the shrimp's stomachs may be an overestimation of the total number of real species (e.g., due to detection of pseudogenes; Tang et al., 2012; Vamos, Elbrecht, & Leese, 2017) and includes protists and microalgae (Wangensteen et al., 2018), which are unlikely to be prey items of *C. crangon*. Nonetheless, even just the 306 ascertained or 20 most abundant (>0.5% relative abundance) species in the shrimp's diet was remarkably higher than the number found in previous studies based on morphological identification (see Table 1). Furthermore, twenty taxa showed a high abundance (>5%) in any given sample, probably representing important prey items at some locations or times.

Two species were predominant in our study: the shore crab *Carcinus maenas* across the overall geographic distribution, and the amphipod *Corophium volutator* in UK localities (characterized by muddy sediments and high organic matter content). Both species are well‐known prey of *C. crangon* (Evans, 1984; Moksnes, Pihl, & van Montfrans, 1998; Pihl & Rosenberg, 1984) and can occur at high densities in soft‐bottom estuarine habitats (Meadows & Reid, 1966; Moksnes, 2002). Consumption of *C. maenas* could be the result of scavenging, although juvenile crabs could be captured, while *C. volutator* are likely to be predated, as these amphipods are small (up to 11 mm TL). Overall, the local distribution of the detected food items followed environmental gradients reflecting their ecology. Euryhaline deposit feeders such as *C. volutator* and *Hediste diversicolor*,* Neomysis integer* and *Chironomus salinarius* (larval stage) were mainly associated with muddy, brackish sites with high organic matter content, commonly inhabited by these species (Anderson, 1972; Drake & Arias, 1995; Mauchline, 1971; Meadows, 1964; Mees, Dewicke, & Hamerlynck, 1993; Ólafsson & Persson, 1986). Stomach samples taken from sites with larger grain size contained species adapted to coarser sands, such as *P. longicornis* and *Talitrus saltator* (Fanini, Marchetti, Scapini, & Defeo, 2007; Pallas, Garcia‐Calvo, Corgos, Bernardez, & Freire, 2006). Detection of fish DNA could reflect a combination of direct predation on juveniles of species which use the estuaries as nurseries (e.g., *Platichthys flesus* and *Dicentrarchus labrax*) and scavenging on dead bodies of species which do not regularly use estuaries as a nursery (e.g., *Scomber scombrus* and *Labrus bergylta*; Elliott & Dewailly, 1995). The high presence of the invasive barnacle *Austrominius modestus* DNA at several locations was likely due to the capture of cyprus or nauplii larvae (Ansell et al., 1999; Boddeke et al., 1986).

This is also the first study showing a high occurrence of *Purpureocillium lilacinum* (Ascomycota: Hypocreales) in the digestive system of *C. crangon*. *P. lilacinum* is a well‐studied fungus, being abundant in terrestrial soils (Cham Thi Mai, Nhi Thi Thuy, Duong Thi Thuy, Hoang Nguyen Duc, & Xo Hoa, 2016) and detected in the marine environment (Redou, Navarri, Meslet‐Cladiere, Barbier, & Burgaud, 2015; Yue et al., 2015). It is a known pathogen of nematodes and therefore of commercial importance as a biological control agent to manage pests of several crops (Castillo Lopez, Zhu‐Salzman, Ek‐Ramos, & Sword, 2014; Singh, Pandey, & Goswami, 2013). This fungus is even considered to be of medical importance since it can infect humans and other vertebrates with compromised immune systems (Luangsa‐Ard et al., 2011). As *P. lilacinum* has been successfully cultured (for the production of chitosanase) using farmed marine shrimp by‐products as substrate (*Penaeus* sp.; Nidheesh, Pal, & Suresh, 2015) and is closely related to known parasites of crabs (Smith et al., 2013), it might be postulated that it has a symbiotic relationship with *C. crangon*, although more research is required to test this hypothesis. Its occurrence and high relative abundance (although possibly overestimated since its DNA was extracted from a living community, as opposed to digested food) in *C. crangon* stomach samples over a large geographical area are clear indicators that this species might be important for the brown shrimp's ecology and/or physiology. Alongside *P. lilacinum*, several other known parasitic taxa have been detected in the shrimp's stomachs, including *Hematodinium* sp. and Apicomplexa (Molnar, Ostoros, Dunams‐Morel, & Rosenthal, 2012; Rueckert, Simdyanov, Aleoshin, & Leander, 2011; Stentiford & Shields, 2005).

### 4.2 The application of metabarcoding in crustacean trophic studies

Metabarcoding using universal primers is generally considered as a simple, rapid and relatively inexpensive method to define in detail the feeding ecology of organisms (Berry et al., 2015; Kartzinel & Pringle, 2015; Pinol et al., 2014). The fraction of the brown shrimp DNA detected in its own gut was low allowing for the detection of prey items without using predator‐specific blocking primers (average: 28%; compared to, e.g., Olmos‐Péerez, Roura, Pierce, Boyer, & González, 2017; Pinol et al., 2014). Metabarcoding has several clear advantages over traditional trophic methods including the better detection of soft‐bodied, small and cryptic taxa, higher speed of analysis (Berry et al., 2015; Casper et al., 2007; Chariton et al., 2015; Symondson, 2002), and traceability of identifications, which do not rely on the availability of morphological taxonomic expertise. Furthermore, the application of metabarcoding even allows for the detection of prey items in empty guts (Harms‐Tuohy, Schizas, & Appeldoorn, 2016), albeit the DNA extracted from visually empty *C. crangon* stomachs was too low in prey read number and MOTU richness for robust comparisons.

Both traditional morphological examination and DNA metabarcoding of food items suffer from limitations in providing quantitative descriptions of the diet of consumers (Casper et al., 2007). For metabarcoding, errors can occur due to technical artefacts specific to DNA amplification and sequencing (Barnes & Turner, 2016; Pompanon et al., 2012), and biological limitations such as species‐specific digestion and DNA degradation rates (Deagle, Chiaradia, McInnes, & Jarman, 2010; Murray et al., 2011; Pinol et al., 2014; Sakaguchi et al., 2017). Furthermore, some of the DNA detected might come from secondary predation (taxa present in the stomach of preyed organisms; Berry et al., 2015; Kartzinel & Pringle, 2015). Cannibalism also imposes a specific problem in trophic molecular studies since it cannot be identified by means of metabarcoding (Berry et al., 2015; Ray et al., 2016). Large brown shrimps are known to be cannibalistic (Evans, 1984; Pihl & Rosenberg, 1984) but the removal of *C. crangon* sequence reads from our data set makes it impossible to gauge insights into the extent of cannibalism in this species. Due to the restrictions in the quantification of consumed prey volume, many trophic studies only use presence/absence data (e.g., Deagle et al., 2010; Harms‐Tuohy et al., 2016; Pinol, Mir, Gomez‐Polo, & Agusti, 2015). This might, however, result in an overestimation of small taxa that are abundant in the sediment, but with low trophic relevance, as they could, in the case of *C. crangon*, be passively acquired when shrimp ingest sediment to crush food in their stomach (Ansell et al., 1999; Deagle et al., 2018; Tiews, 1970). Multiple stomachs were pooled prior to analysis and data were subjected to rigorous filtering to allow for a semiquantitative estimation of proportions of prey DNA (Deagle et al., 2005; Lejzerowicz et al., 2015; Pompanon et al., 2012; Thomas, Deagle, Eveson, Harsch, & Trites, 2016). Relative abundances of individual taxa should, however, be considered with caution and viewed more in categorical terms (low or high trophic significance) than exact proportions (Deagle et al., 2005). This study provides a significant addition to a growing body of studies in showing the applicability of semiquantitative estimations in molecular trophic ecology (e.g., Albaina et al., 2016; Deagle et al., 2018; Ray et al., 2016; Sakaguchi et al., 2017; Soininen et al., 2013).

Finally, the results presented draw a close link between prey distribution in estuarine habitats and ingested prey item abundance. The use of eDNA from sediments to assess community composition and to relate this to the shrimp's diet is a novel contribution to the fields of molecular trophic analysis and eDNA, which goes beyond the taxon studied. It should be noted, however, that a correct assessment of the predator's trophic niche by means of prey selectivity determination relies on a correct assessment of prey abundance, both in the stomach and in the environment. Issues with incorrect abundance estimations, for example due to species‐specific detection rates (e.g., due to different rates of DNA sequestering in the environment; Barnes & Turner, 2016), are not specific to molecular studies (Strauss, 1979) and the constant work on improving the reliability of relative abundance estimations from eDNA (Deagle et al., 2018; Thomas et al., 2016; Ushio et al., 2018) should substantially enhance the applicability of selectivity indices in molecular research.

### 4.3 Geographic variation in *C. crangon* trophic ecology

This study also assesses for the first time a large geographical variation in the brown shrimp's trophic ecology at multiple spatial scales. Previous studies have shown local variability in *C. crangon* diet (Evans, 1984; Oh et al., 2001; Pihl & Rosenberg, 1984) but no studies have been performed across multiple European estuaries. The results indicate that the consumed prey community can vary at local (within estuary, as discussed above) and regional (between estuaries) scales. The seasonal and tidal migratory behaviour of *C. crangon* (Al‐Adhub & Naylor, 1975; Henderson & Holmes, 1987) may complicate localized diet assessments since their stomach contents might also contain food consumed at distant locations. Yet, this effect is considered to be minimal since the brown shrimp's relatively fast gut passage time ensures that their stomach contents mainly contain recently consumed items (4–20 hr; Feller, 2006; Pihl & Rosenberg, 1984; van der Veer & Bergman, 1987). Large‐scale assessment of *C. crangon*'s trophic ecology showed high similarity between the Eastern Scheldt, Western Scheldt and Mersey estuaries and distinct diets in the Aveiro, Minho and Kent estuaries. The Aveiro Ria forms a large, saline lagoon with a wide variety of different habitats incorporating euryhaline/polyhaline areas with relatively high species richness (Rodrigues, Quintino, Sampaio, Freitas, & Neves, 2011). On the other hand, the Minho estuary is characterized by high water discharge, salinity variations (Costa‐Dias, Freitas, Sousa, & Antunes, 2010) and significantly larger sediment grain size, factors which determine significantly divergent biodiversity features. The Kent estuary has a low species diversity caused by its fine sediments and low salinity (Anderson, 1972). The Mersey estuary also showed a relatively low species richness detected in the stomach contents of *C. crangon*, probably related to its history of anthropogenic stress (Jones, 2000). Overall, trophic variation in *C. crangon* depends on patterns in the local abundance and distribution of its prey (in line with: Oh et al., 2001; Pihl, 1985; Pihl & Rosenberg, 1984). In order to evaluate this variation more exhaustively, knowledge on the ecology and seasonality of the local macrozoobenthic community is required.

### 4.4 *Crangon crangon*'s ecological role

**Figure 8:**
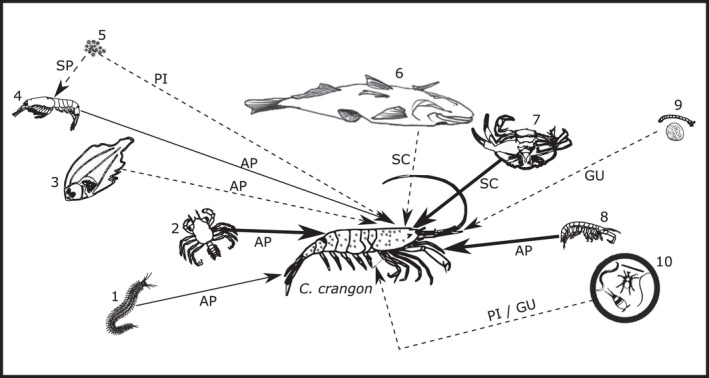
Schematic representation of the most important food items of adult *Crangon crangon* and their probable method of capture/ingestion. Line thickness represents trophic significance: high (bold); medium (thin); low (dashed). Numbers identify prey categories: annelids (1); decapod larvae/instars (2); fish 0‐year‐juveniles (3); 4 mysids (4); (pico) phytoplankton (5); fish carcasses (6); decapod carcasses (7); amphipods (8); chironomid, mollusc and barnacle larvae (9); meiofauna (10). Letters define method of ingestion: Secondary predation (SP); ambush predation (AP); gulping predation (GU); passive ingestion (PI); scavenging (SC). Images not to scale

Based on the results of this study, *C. crangon* can best be described as a highly opportunistic carnivore and scavenger. Despite its broad dietary range (Figure 8), it shows a prominent level of selectivity for larger mobile epifaunal prey items. This high level of flexibility in its trophic ecology might contribute to its very wide distribution on European coasts (Campos et al., 2009). In order to feed on diverse prey taxa, adult *C. crangon* are capable of employing a variety of methods (Figure 8) including (camouflage‐assisted) ambush predation (Gibson et al., 1995; Pinn & Ansell, 1993; Siegenthaler, Mastin, Dufaut, Mondal, & Benvenuto, 2018), gulping behaviour (Tiews, 1970) and scavenging (Figure 8; Ansell et al., 1999; Price, 1962). Since meiofaunal and protist phyla were not selected as prey items based on Jacobs’ selectivity index (present but not abundant; in line with: Evans, 1983; Feller, 2006), it is possible that these taxa were passively consumed during the ingestion of sand to aid digestion (Ansell et al., 1999; Tiews, 1970) or through secondary predation. Several studies classify *C. crangon* as an omnivore (Ansell et al., 1999; Raffaelli et al., 1989; Tiews, 1970), but we cannot confirm this classification, because the primers used during this study have a very low affinity for chlorophytes resulting in many algal taxa not being detected (Wangensteen et al., 2018). Nevertheless, the algal phyla that can be detected with these primers (e.g., Rhodophyta, Phaeophyta and Bacillariophyta) had a low selectivity, indicating a negligible trophic importance for *C. crangon*. More research is required with plant‐specific primers to assess the actual contribution of herbivory to the diet of the brown shrimp. Overall, the results of this study yield a level understanding of the trophic ecology of this species that would not have been possible through traditional morphological analysis, and which is key to providing essential insights into coastal community interactions.

## AUTHOR CONTRIBUTION

A.S. participated in the study design; carried out the field, laboratory and molecular work; performed the data and statistical analyses; and wrote the manuscript; O.S.W. participated in the molecular work, designed the bioinformatics pipeline and assisted in the statistical analyses; C.B. participated in the study design; J.C. assisted in the collection of the samples; and S.M. conceived and designed the study. All authors provided critical comments on the manuscript and gave final approval for publication.

## Data Availability

The data set, including sequences, taxonomic assignment and abundances for all MOTUs in every sample, has been deposited in Dryad (https://doi.org/10.5061/dryad.sk2155m). Custom R scripts are publicly available from https://github.com/Andjin/Crangon-diet-analysis and https://github.com/metabarpark for scripts related to the bioinformatics pipeline.
