# Supplementary material for: DNA metabarcoding unveils multiscale trophic variation in a widespread coastal opportunist
Source: Mol Ecol. 2018 Oct 24;28(2):232–49. doi: 10.1111/mec.14886 (PMC7380037; doi:10.1111/mec.14886)
Supplement: Supplementary file 1 [file MEC-28-232-s001.pdf]

MOLECULAR ECOLOGY

Supplemental Information for:

DNA metabarcoding unveils multi-scale trophic variation in a widespread coastal opportunist

Andjin Siegenthaler, Owen S. Wangensteen, Chiara Benvenuto, Joana Campos & Stefano Mariani

Table of Contents:

**Table S1**	Page 2
**Table S2**	Page 3
**Table S3**	Page 4
**Table S4**	Page 5
**Figure S1**	Page 6
**Table S5**	Page 7
**Table S6**	Page 8
**Table S7**	Page 9

MOLECULAR ECOLOGY

Table S1. Number of pooled full *Crangon crangon* stomachs per sample. Only samples with less than 8 pooled stomachs are shown. * Samples not used for molecular analysis (< 1000 diet-related reads)

Estuary	Sample	N stomachs
Aveiro	Av_1C	4
Aveiro	Av_2B	7
Eastern Scheldt	ES_2A	5
Eastern Scheldt	ES_2B*	4
Eastern Scheldt	ES_3C	5
Kent	Ke_1C*	5
Kent	Ke_4C	7
Mersey	Me_1C	4
Mersey	Me_2A*	6
Mersey	Me_3B*	7
Western Scheldt	WS_2C	4
Western Scheldt	WS_4C	4

MOLECULAR ECOLOGY

Table S2. Mean (±SD) total length of *Crangon crangon* (>20 mm TL) sampled and dissected for stomach DNA extraction per site. * Site not used for molecular analysis

		Sampled		DNA extracted	
**Estuary**	**Site**	**Mean (mm)**	**SD**	**Mean (mm)**	**SD**
Aveiro	Av_1	32	5	32	6
Aveiro	Av_2	30	4	30	3
Aveiro	Av_3	31	4	31	5
Eastern Scheldt	ES_1	25	6	27	6
Eastern Scheldt	ES_2	33	7	32	6
Eastern Scheldt	ES_3	30	6	32	6
Eastern Scheldt	ES_4	34	7	34	8
Kent	Ke_1	32	3	31	3
Kent	Ke_2	32	3	32	4
Kent	Ke_3	31	3	32	3
Kent	Ke_4	29	2	29	2
Mersey	Me_1	47	4	48	3
Mersey	Me_2	40	7	42	7
Mersey	Me_3*	40	7		
Mersey	Me_4	39	7	36	5
Mersey	Me_5	41	7	40	6
Mersey	Me_6	42	7	40	4
Minho	Mi_1	41	4	39	3
Minho	Mi_2	39	5	38	4
Minho	Mi_3	35	5	36	3
Western Scheldt	WS_1	32	5	32	4
Western Scheldt	WS_2	26	4	27	4
Western Scheldt	WS_3	36	6	37	6
Western Scheldt	WS_4	44	6	43	7

MOLECULAR ECOLOGY

Table S3. *Purpureocillium lilacinum* sequences detected in *Crangon crangon* stomach samples

Marker	Sequence	Match	Query cover	E-Value	Identity
COI	TTTATCAGGATTACAAAGTCACAGTGGACCTA<br>GTGTAGATTTAGCAATTTTTGCTTTACACCTTT<br>CAGGGGTAAGTAGTTTATTAGGAGCAATAAA<br>CTTCATAACTACAATCGCTAATATGAGAACAC<br>CAGGAATAAGATTACACAAATTAGCCTTATTC<br>GGGTGAGCTGTAGTTATAACAGCTATCTTATT<br>ATTATTATCATTACCTGTTTTAGCTGGAGGTAT<br>TACAATGGTATTAACAGATAGAAATTTTAACA<br>CTTCATTCTTCGAAGTAGCTGGTGGTGGAGAT<br>CCTATATTATTCCAACACTTATTC	*Purpureocillium lilacinum*	100%	$2e^{-156}$	99%
ITS	ACTCCCAAACCCACTGTGAACCTTACCTCAGTT<br>GCCTCGGCGGGAACGCCCCGGCCGCCGGCCC<br>CCGCGCCGGCGCCGGACCCAGGCGCCCGCCG<br>CAGGGACCCCAAACTCTCTTGCATTACGCCCA<br>GCGGGCGGAATTTCTTCTCTGAGTTGCACAAG<br>CAAAAACAAATGAATCAAAACTTTCAACAACG<br>GATCTCTTGGTTCTGGCATCGATGAAGAACGC<br>AGCGAAATGCGATAAGTAATGTGAATTGCAG<br>AATTCAGTGAATCATCGAATCTTTGAACGCAC<br>ATTGCGCCCGCCAGCATTCTGGCGGGCATGCC<br>TGTTCGAGCGTCATTTCAACCCTCGAGCCCCCC<br>CGGGGGCCTCGGTGTTGGGGGACGGCACACC<br>AGCCGCCCCCGAAATGCAGTGGCGACCCCGC<br>CGCAGCCTCCCCTGCGTA	*Purpureocillium lilacinum* isolate NIOSN_SK56_S76	100%	0.0	100%

MOLECULAR ECOLOGY

Table S4. List of pooled stomach samples removed prior to analysis due to low read depth (< 1000 reads) after removal of predator, parasite and contaminant reads. * Removal of sediment sample due to lack of site Me_3 stomach samples included in the final analysis.

Estuary	Sample	Number of reads
Western Scheldt	WS_1B	330
	WS_3B	486
	WS_4A	420
Eastern Scheldt	ES_1A	396
	ES_2B	897
	ES_3A	214
	ES_4A	538
	ES_4C	366
Mersey	Me_2A	364
	Me_3A	179
	Me_3B	486
	Sediment sample Me_3*	27212
Kent	Ke_1C	669
	Ke_2A	189
	Ke_4A	301

MOLECULAR ECOLOGY

Figure S1. Rarefaction curves of the number of MOTUs obtained at increasing numbers of reads in the sediment (A) and stomach (B) samples per site. *Crangon crangon* and *Purpureocillium lilacinum* have been removed from the stomach samples prior to analysis.

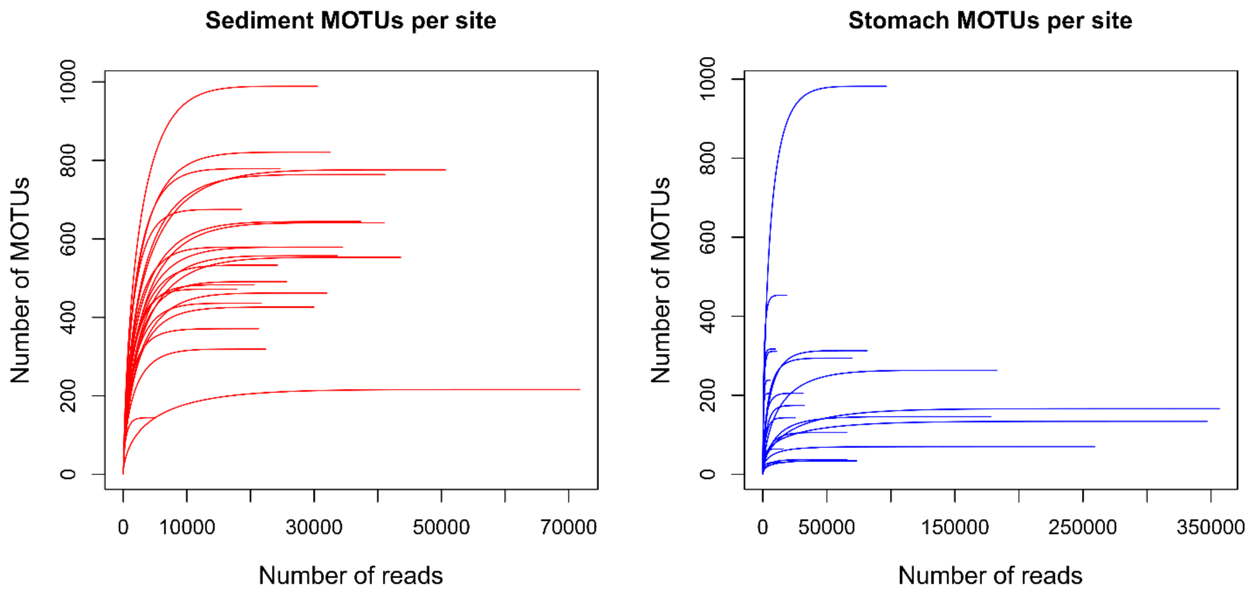

MOLECULAR ECOLOGY

Table S5. Differences in relative mean read abundance of detected phyla in sediment and *Crangon crangon* full stomach samples. Samples are averaged per site (N = 23) and tested with a paired Wilcoxon signed-rank test. **: P < 0.01

	Mean relative abundance (%)		Wilcoxon signed-rank test		
**Phylum**	**Sediment**	**Full Stomach**	**W**	**P**	
Annelida	3.43	16.12	225	0.007	**
Arthropoda	5.30	48.56	274	0.000	**
Bacillariophyta	20.47	1.69	0	0.000	**
Chordata	0.08	4.81	208	0.000	**
Cnidaria	0.79	1.08	100	0.259	
Dinoflagellata	6.13	1.55	53	0.008	**
Discosea	10.40	0.20	0	0.000	**
Mollusca	0.16	1.18	192	0.001	**
Oomycota	2.87	0.31	3	0.000	**
Rhodophyta	2.00	1.71	111	0.427	
Unassigned	44.01	18.92	9	0.000	**

MOLECULAR ECOLOGY

Table S6. Pairwise comparisons (Bonferroni-corrected) of square-root transformed Bray-Curtis dissimilarities of *Crangon crangon* pooled stomach contents between estuaries. * = P < 0.05; ** = P < 0.01.

Pairs	F.Model	Df	R^2	P	
Eastern Scheldt vs WScheld	1.684	1,12	0.123	1.000	
Eastern Scheldt vs Minho	3.648	1,13	0.219	0.046	*
Eastern Scheldt vs Aveiro	2.948	1,12	0.197	0.460	
Eastern Scheldt vs Mersey	2.782	1,17	0.141	0.105	
Eastern Scheldt vs Kent	9.698	1,12	0.447	0.010	*
Western Scheldt vs Minho	2.368	1,15	0.136	0.426	
Western Scheldt vs Aveiro	1.872	1,14	0.118	0.784	
Western Scheldt vs Mersey	1.535	1,19	0.075	1.000	
Western Scheldt vs Kent	7.116	1,14	0.337	0.015	*
Minho vs Aveiro	4.914	1,15	0.247	0.015	*
Minho vs Mersey	3.021	1,20	0.131	0.015	*
Minho vs Kent	9.597	1,15	0.390	0.001	**
Aveiro vs Mersey	4.152	1,19	0.179	0.007	**
Aveiro vs Kent	13.336	1,14	0.488	0.004	**
Mersey vs Kent	4.224	1,19	0.182	0.030	*

MOLECULAR ECOLOGY

Table S7. Mean (± SD) Salinity, median grainsize and total organic matter (TOM) per estuary

**Estuary**	**Salinity**	**Grain Size (μm)**	**TOM (%)**
Aveiro	25.5 (± 8.5)	271 (± 151)	1.32 (± 0.96)
Eastern Scheldt	31 (± 0.2)	294 (± 85)	0.28 (± 0.11)
Kent	0.3 (± 0.1)	76 (± 6)	0.92 (± 0.35)
Mersey	17.6 (± 10.2)	132 (± 90)	2.6 (± 3.47)
Minho	6.5 (± 3.5)	477 (± 181)	0.99 (± 1.15)
Western Scheldt	23.2 (± 5.6)	259 (± 101)	0.81 (± 0.57)
